# Motivational climate in school sport and adolescent mental health: a systematic review

**DOI:** 10.3389/fpsyt.2026.1907748

**Published:** 2026-09-03

**Authors:** Yuan Wei, Guan-Nan Lai, Cheng Ji, Xiao-Ming Yang, Chun-Yuan Tie

**Affiliations:** 1 Department of Sports Studies, Faculty of Educational Studies, Universiti Putra Malaysia, Kuala Lumpur, Selangor, Malaysia; 2 College of Physical Education, Putian University, Putian, China; 3 School of Sport Science, Qufu Normal University, Jining, China; 4 School of Business, Beijing Technology and Business University, Beijing, China

**Keywords:** adolescent mental health, motivational climate, physical education, school sport, systematic review

## Abstract

**Background:**

Motivational climate in school-based physical education and sport represents a potentially modifiable context for adolescent mental health; however, its associations with psychological outcomes have not been systematically synthesised. This review aimed to synthesise evidence on perceived motivational climate and adolescent mental health outcomes in school settings.

**Methods:**

Databases were searched from the inception to May 2026 following PRISMA guidelines. Inclusion criteria for studies included sampling of adolescents (10–19 years of age), assessment of motivational climate in school-based sport or physical education, reporting of psychological outcomes, and quantitative or mixed-method designs with original data. The JBI critical appraisal tools were used to appraise methodological quality. Findings were narratively synthesised because of the heterogeneity of the study designs, measurements and outcomes.

**Results:**

A total of 19 studies were included with a total of 17, 825 adolescents across 8 countries. The quality of sixteen studies was high and three were rated as moderate. Across the included studies, task-involving, caring and empowering climates were generally associated with positive mental health outcomes such as subjective well-being, self-esteem, enjoyment, positive affect, and social-emotional competence. However, climates that were ego-involving and disempowering were associated with higher anxiety, depression, perceived stress, and shame. Multiple psychological, relational, cognitive, and behavioural explanatory pathways were identified, including basic psychological need satisfaction, intrinsic motivation, teacher-student and peer relationships, deliberate rumination, and physical education engagement.

**Conclusions:**

The available evidence suggests that task-involving, caring, and empowering climates in school sport are generally associated with better adolescent mental health, whereas ego-involving and disempowering climates are associated with more anxiety, depression, stress, and shame. These associations may be partially explained by more than just basic psychological need satisfaction. Intrinsic motivation, supportive teacher-student and peer relationships, and even deliberate rumination also play a role. In short, how the sport climate meets adolescents’ psychological and social needs matters. Although causal inferences are precluded by the predominantly cross-sectional design of the included studies, shifting PE away from rigid competition and towards effort and mutual support could represent a practical approach to protect youth mental health.

**Systematic review registration:**

https://www.crd.york.ac.uk/prospero/, identifier CRD420261419406.

## 1 Introduction

Mental health issues in adolescence represent a significant proportion of disease burden worldwide and often have a life-long impact (1). Schools are identified as key settings for mental health promotion because of the structured social and emotional learning opportunities they provide (2, 3). In particular, physical education and school sport may influence adolescent psychological well-being by integrating physical activity, peer interaction, and evaluative experiences within a structured educational environment (4).

A key environmental feature in these settings is the motivational climate—the achievement-related cues, expectations, and evaluative practices perceived by students (5). Grounded in Achievement Goal Theory (AGT), motivational climates are commonly distinguished as task-involving (emphasizing effort, learning, and personal improvement) versus ego-involving (emphasizing normative comparison and outperforming others) (6). Self-Determination Theory (SDT) has subsequently been integrated to explain the mediating mechanisms: basic psychological need satisfaction has been proposed as an important explanatory pathway linking motivational climate with psychological functioning and well-being (7). More recent frameworks have added caring climates (emotional support and mutual respect) and the empowering–disempowering continuum (autonomy support versus coercion and control) (8). Accumulated evidence suggests that task-involving, caring, and empowering climates are associated with adaptive outcomes such as higher intrinsic motivation and self-esteem, whereas ego-involving and disempowering climates are associated with anxiety, shame, and disengagement (9). However, these findings are dispersed across diverse theoretical frameworks, operationalisations, and outcome domains, limiting the extent to which an integrated understanding can be achieved.

To our knowledge, no previous systematic review has specifically examined the associations between motivational climate within school-based physical education and sport settings and a comprehensive range of adolescent mental health outcomes. The syntheses that have been conducted have focussed on general school climate (10), without isolating motivational climate as a specific environmental construct (11), or physical activity in general (12). Meta-analyses of motivational climate have either aggregated diverse outcomes without specifically examining mental health (13) or examined competitive sport populations (14), thus not specifically targeting mental health in school settings. Therefore, existing evidence regarding the associations between different motivational climate dimensions (e.g., task-involving, ego-involving, caring, empowering, and disempowering climates) and diverse adolescent mental health outcomes in school-based physical education and sport contexts remains fragmented and has not been systematically synthesised. In addition, potential explanatory factors (e.g., basic psychological need satisfaction, intrinsic motivation, and teacher-student or peer relationships) have been examined inconsistently across individual studies and remain insufficiently synthesised.

Beyond school-based sport contexts, evidence from structured physical activity environments provides additional contextual insights into the potential relationship between organised activity experiences and psychological functioning. For example, structured taekwondo training has been associated with self-control and quality-of-life outcomes amongst sedentary women (35), whilst recreational yoga participation has been linked with flow experiences and perceived health outcomes (36). These studies, however, were done with adults and so offer indirect contextual evidence, but not direct evidence relevant to the context of school based adolescent mental health. However, they offer a complementary perspective on the psychological regulation, subjective well-being and experiential processes to consider when the possible effects of physical activity environments on psychological functioning are considered.

To fill such gaps, the aim of this systematic review was to provide a synthesis of empirical evidence concerning relations between perceived motivational climate in school based physical education and sport settings and adolescent mental health outcomes and to explore possible psychological and social mechanisms reported in the literature.

## 2 Methods

### 2.1 Eligibility criteria

Systematic review performed and reported according to Preferred Reporting Items for Systematic Reviews and Meta-Analyses (PRISMA) 2020 statement (37). Studies were included that fulfilled the following PICOS criteria.

Population: School going adolescents, approximately 10–19 years old. Studies that targeted adult populations, children or non-school populations were excluded. Exposure: Motivational climate as perceived in school affiliated PE/sport settings (PE classes, school sport teams, organised school based sport). Only studies set in community/sport club or non-school environments were included. No restriction was made: Comparison. Observational studies were included as well as comparative studies that contrasted the levels of or types of motivational climates. Outcome: Any mental health indicator such as depression, anxiety, perceived stress, shame, self-esteem, subjective well-being, school belonging, social-emotional competence, positive affect/enjoyment, coping appraisals. Studies not having a mental health variable, but only physical health or academic outcomes were excluded. Consistent with the WHO definition of mental health as a state of well-being encompassing coping abilities and positive functioning (33), these outcomes were included as they reflect psychological well-being and adaptive functioning alongside traditional clinical indicators. Study design: Quantitative or mixed-methods studies with original data, published in peer-reviewed English journals. Reviews, meta-analyses, conference abstracts, editorials, dissertations, and non-peer-reviewed articles were excluded. Studies with insufficient data or unavailable full text were also excluded.

### 2.2 Data sources and search

We conducted a systematic literature search to identify studies examining the relationship between school sport climate and adolescent mental health. Searches were performed across major bibliographic databases and relevant academic platforms, including Web of Science, PubMed, and EBSCOhost (including SPORTDiscus, PsycINFO, and ERIC), from their respective inception dates through May 2026. Searches of relevant publisher platforms, including Sage Journals and Springer Nature Link, were also conducted to maximise the retrieval of potentially eligible studies. This review was registered on PROSPERO (ID: CRD420261419406; registration date: 09 June 2026) and was reported in accordance with the PRISMA 2020 statement. The review followed the predefined eligibility criteria and methodological framework specified in the registered protocol.

Based on the three ideas of motivational climate, school-based physical education and sports environments, and mental health outcomes, the search strategy has been developed. Boolean operators (AND, OR) are used to combine the search terms from the three categories: Motivational climate (e.g., task-oriented, mastery-oriented, ego-oriented, caring, empowering, disempowering climate), School sport contexts (e.g., physical education, school sports, school athletics, athletic teams), and Mental health indicators (e.g., well-being, social-emotional competence, self-esteem, anxiety, depression, stress, psychological distress). Database-specific search syntax was employed when necessary, and all the search strategies are provided in Supplementary Material S1. Backward and forward citations were carried out, and Google Scholar was also employed as an extra tool to find some relevant studies.

### 2.3 Study selection

All the obtained records were imported into the reference management software EndNote X9 and duplicates were removed. Two people (YW and CJ) were chosen by themselves to do so. The first step is to check whether the title and abstract of all items meet the prerequisites. Then, related full-text articles were retrieved and independently judged by the same two reviewers. Any disputes during the screening and full-text review stages will be settled by discussion; if no agreement can be reached, a third reviewer (GL) will be consulted. The general selection was overseen and judged by the corresponding author (CT). The particular reasons for excluding the full-text articles have been recorded and shown in the PRISMA flow diagram.

### 2.4 Data extraction

According to the standardised and pilot-tested data collection form, the data will be collected regularly. The two reviewers independently collected the corresponding materials from all the qualified studies. For the purpose of this study, the variables obtained from the data are as follows (1): Study characteristics (author, year, country and type of study) (2); Participant information (sample size, age and gender distribution) (3); Exposure to the sport climate and changes in mental health, along with their corresponding measurement tools and sub-dimensions; and (4) Main statistical results. Any differences in the two extracted results were resolved through discussion amongst them, or a third party was consulted. If there is any missing or incomplete data, the corresponding author has contacted the primary authors of that paper via email to request clarification.

### 2.5 Quality assessment

The two reviewers independently judged the quality of the methods in the included studies according to the standard assessment tools provided by the Joanna Briggs Institute (JBI). Specifically, the analysis of cross-sectional studies was carried out according to the JBI Checklist for Analytical Cross-Sectional Studies (15), quasi-experimental studies were evaluated based on the JBI Checklist for Quasi-Experimental Studies (16), and the longitudinal cohort study was judged according to the JBI Checklist for Cohort Studies. Each item was answered with “Yes” (Y), “No”(N), “Unclear” (U) or “Not applicable” (NA). Studies were categorised as high quality (uali of applicable items rated as “stedca moderate quality (50lityel or low quality (<50%). Although the JBI Manual for Evidence Synthesis does not specify universal cut-off points for quality classification, this predefined percentage-based approach has been widely used in JBI-informed systematic reviews to facilitate transparent interpretation of methodological quality (17). The two reviewers could not agree, so a third person was asked to mediate and resolve the dispute.

### 2.6 Data synthesis

Due to the high level of heterogeneity between studies in terms of study designs, motivational climate constructs and mental health outcomes, including varied measurement instruments (e.g., PMCSQ-2, TEOSQ), study designs (cross-sectional vs. longitudinal), and outcome scales (e.g., CDI, SCARED, SDQ), a quantitative meta-analysis was not deemed appropriate. Effect size conversion was considered but precluded by inconsistent reporting formats (e.g., β coefficients, Cohen’s d); subgroup meta-analyses were also explored but not pursued due to limited study counts and persistent measurement heterogeneity. Hence, a narrative synthesis was performed as per the guidelines of Synthesis Without Meta-analysis (SWiM) (18).

Results were narratively synthesised according to four conceptually distinct mental healthctallyMA domains: (1) negative psychological indicators, including depression, anxiety, stress, shame, and negative affect; (2) positive mental wellalveon, encompassing subjective wellectiven selfectiveng and positive affect; (3) affective and motivational outcomes, comprising enjoyment, pleasure, and functional psychobiosocial states; and (4) psychosocial and developmental competencies, including coping appraisals, physical selficalls,s, socialalls,s,al competence, and life skills. Based on the relationships between motivational climate dimensions and mental health outcomes, findings were interpreted whilst accounting for contextual factors such as country, age, gender composition and sport setting.

## 3 Results

### 3.1 Study selection

A systematic search was performed and a total of 582 records were found in the databases included in the search. All records were then imported to EndNote X9 and 27 were found to be duplicates and these were deleted. Of the 91 records excluded by predefined filters in the databases (e.g., publication type, subject area, and population-related filters), 90 were excluded because they did not meet the inclusion criteria and 1 record was excluded because it was a duplicate. These exclusions were then double checked by two independent reviewers to avoid the possible loss of potentially eligible studies. After the above process, there were 464 records left for title and abstract screening.

Following the title and abstract screening, 405 records were excluded as they failed to meet the PICOS eligibility criteria, mainly because they included non-adolescent populations, non-school sport settings, or no motivational climate constructs, or lacked appropriate mental health outcomes. 59 full-text articles were evaluated for eligibility, with one article not being able to be retrieved despite efforts via interlibrary loan or direct contact with the authors. Of the remaining 58 full-text articles 39 were excluded because they did not identify a valid measure of the motivational climate of school sports or measure mental health outcomes in adolescents.

**Figure 1:**
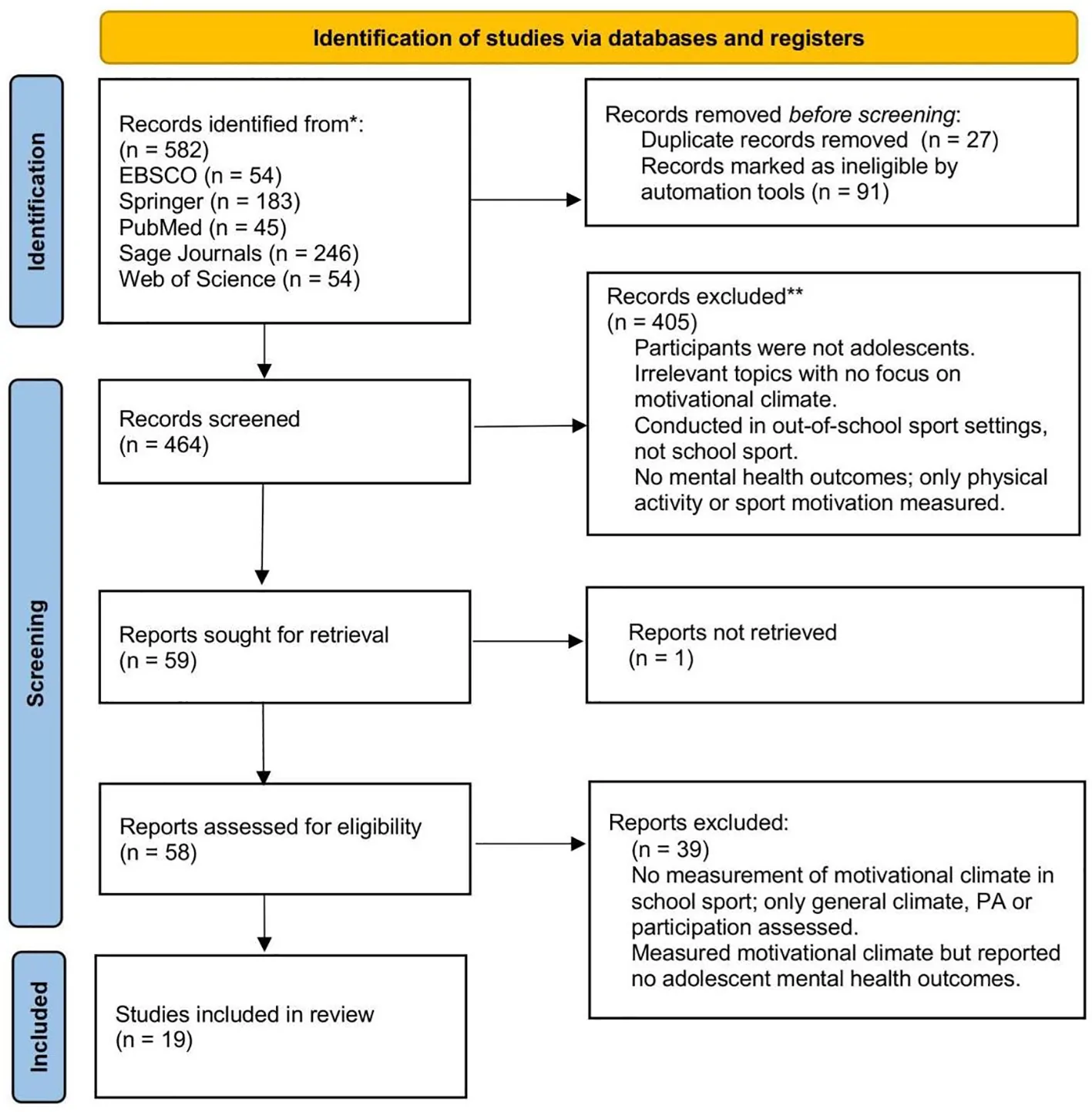
PRISMA flow diagram of study selection for the systematic review of motivational climate and adolescent mental health.

Finally, 19 original studies fulfilled all the PICOS inclusion criteria and were included in this systematic review. The selection process of the studies is presented by creating a PRISMA 2020 flow diagram (**Figure 1**).

### 3.2 Quality assessment results

The methodological quality of the 19 included studies is summarised in **Table 1**. Amongst the 14 cross-sectional studies, twelve were rated as high quality and two as moderate quality. Of the 3 quasity.ctionallts studies, all received a “ecei on 8 or 9 items, indicating high quality. Of the two cohort studies, one was high quality and the other moderate quality. Overall, 16 studies were classified as high quality and three as moderate quality; no study was rated as low quality.

**Table 1:** Critical appraisal of included studies using JBI checklists.

Authors (year)	Country	Study design	JBI ratings	Total	Quality level
Cheng & Jiao (2024) (23)	China	Cross-sectional	YYYYYYYY	8	High
Hogue et al. (2018) (8)	USA	Cross-sectional	YYYYYYYY	8	High
Hogue (2024) (21)	USA	Cross-sectional	YYYYYYYY	8	High
Massey et al. (2025)	USA	Cross-sectional	YYYYYYYY	8	High
Jaakkola et al. (2015) (19)	Finland	Longitudinal Cohort	YUYYYYYYUUY	8	High
Castro-Sánchez et al. (2019) (6)	Spain	Cross-sectional	YYYNNYYY	6	High
Zhao et al. (2026) (29)	China	Cross-sectional	YYYNNNYY	5	Moderate
Granero-Gallegos et al. (2026) (9)	Spain	Cross-sectional	YYYNYYYY	7	High
Xue et al. (2026) (27)	China	Cross-sectional	YYYNYYYY	7	High
Liukkonen et al. (2010) (20)	Finland	Cross-sectional	YYYNYYYY	7	High
Bortoli et al. (2015) (25)	Italy	Quasi-experimental	YYYYYYYYY	9	High
Taylor et al. (2014) (31)	UK	Longitudinal	UUYYYUYYUYY	7	Moderate
Huhtiniemi et al. (2021) (32)	Finland	Cross-sectional	YYYYNNYY	6	High
Mastagli et al. (2021) (22)	France	Cross-sectional	YYUYNNYY	5	Moderate
Simon et al. (2025) (24)	France	Cross-sectional	YYYYNNYY	6	High
Barkoukis et al. (2010) (38)	Greece	Quasi-experimental	YNYYYYYYY	8	High
Li et al. (2024) (39)	USA	Quasi-experimental	YNYYYYYYY	8	High
Coterón López et al. (2013) (40)	Spain	Cross-sectional	YYYNYYYY	7	High
Vlachos et al. (2025) (41)	Greece	Cross-sectional	YYYNYYYY	7	High

Appraised by JBI checklists (cross-sectional 8 items, quasi-experimental 9 items, cohort 11 items). Y, Yes; U, Unclear; N, No.

### 3.3 Characteristics of included studies

In total, 19 studies were identified for this review from the years 2010 to 2026 (**Table 2**). Studies were carried out in eight countries: China (3), the United States (4), Finland (3), Spain (3), France (2), Greece (2), Italy (1) and the United Kingdom (1). Fourteen studies used cross sectional design and 2 studies used longitudinal design and 3 studies used quasi experimental design. The studies included in this review had a combined total of 17, 825 adolescents as participants in physical education/sport activities in school settings. The sample size was between 108 and 2, 956 participants, and the age span was approximately 10–19 years, in keeping with the definition of an adolescent population.

**Table 2:** Characteristics of included studies focused on motivational climate and adolescent mental health indicators.

Author (year)	Country	Sample (N, %female, age, years)	Motivational climate measure	Mental health outcome	Measurement tool	Key findings
Cheng & Jiao (2024) (23)	China	1,666; 51.2%; 10–15 years	Campus Sports Atmosphere Scale	Subjective well-being	Campbell Happiness Index	Sports atmosphere positively predicted subjective well-being.
Hogue et al. (2018) (8)	USA	349; 52.1%; 15.69 ± 1.29 years	PMCEQ-A; CCS	Life stress; Shame; Coping appraisals	PSS; ISS; PASA	Task/Caring climates enhanced coping appraisals and reduced shame. Ego climate increased life stress and shame for both genders.
Hogue (2024) (21)	USA	181; 71.3%; 15.71 ± 1.28 years	CCS; PMCSQ	Depression; Life stress; Psychosocial stress; Coping appraisals; Enjoyment	BDI-PC; PSS; ISS; PASA	Caring climate reduced depression, life stress, and shame. Task climate increased coping appraisals and enjoyment.
Massey et al. (2025) (25)	USA	2,956; 100%; 12–17 years	PNSS-S; MCSYS; CART-Q; YSEQ	Depression; Anxiety	PROMIS	Ego climate increased depression/anxiety. Autonomy support, coach closeness, and social cohesion reduced depression/anxiety.
Jaakkola et al. (2015) (19)	Finland	540; 48.7%; 12–14 years	MCPEQ	PE enjoyment	Sport Enjoyment Scale	Learning climate directly and indirectly predicted higher PE enjoyment.
Castro-Sánchez et al. (2019) (6)	Spain	2,452; 57.3%; 13–16 years	PMCSQ-2	Life stress	PSS	Task climate reduced life stress for both genders; ego climate increased life stress for both genders.
Zhao et al. (2026) (29)	China	1,145; 51.3%; 13.99 ± 1.08 years	EDMCQ-PE	Social-emotional competence	DSECS-S	Empowering climate promoted social-emotional competence (mediated by teacher-student relationships & physical activity).
Granero-Gallegos et al. (2026) (9)	Spain	2,438; 50.7%; 16.15 ± 2.01 years	EDMCQ-PE	Self-esteem	Rosenberg Self-Esteem Scale	Empowering climate increased self-esteem via need satisfaction; disempowering climate reduced self-esteem.
Xue et al. (2026) (27)	China	549; 52.1%; 15.17 ± 1.55 years	PE-EMCS	Life skills	Chinese version of Life Skills Scale for PE	Empowering climate fostered life skills directly and indirectly via deliberate rumination and PE engagement.
Liukkonen et al. (2010) (20)	Finland	338; 48.2%; 11–12 years	MCPEQ	Enjoyment; State anxiety	Sport Enjoyment Scale; PESAS	Task climate and autonomy/relatedness increased enjoyment. Ego climate increased anxiety and reduced enjoyment.
Bortoli et al. (2015) (25)	Italy	108; 100%; 14–15 years	TIMCPEQ	Pleasant/functional PBS states; Unpleasant/dysfunctional PBS states	PBS	Ego climate intervention decreased perceived task climate/pleasant states, and increased perceived ego climate/unpleasant states.
Taylor et al. (2014) (31)	UK	545; 51%; 10.82 ± 0.39 years	TASCQ; Peer MCYSQ	Physical self-concept	PSDQ	Teacher need support and autonomous motivation positively predicted stable physical self-concept over time.
Huhtiniemi et al. (2021) (32)	Finland	645; 50%; Grade 5/8 (M = 11.2/14.2 years)	MCPES	Enjoyment; Anxiety	SCQ-2; PESAS	Task climate increased enjoyment and decreased anxiety. Ego climate increased anxiety.
Mastagli et al. (2021) (22)	France	425; 66.1%; 15.36 ± 0.82 years	EMCS	Positive affect; Negative affect	PANAS	Empowering climate increased positive affect and reduced negative affect via competence need satisfaction.
Simon et al. (2025) (24)	France	350; 56.0%; 15.67 ± 0.87 years	EDMCQ-PE	Pleasure; State anxiety	STAI; PACES	Empowering climate increased pleasure via need satisfaction and reduced anxiety. Disempowering climate increased anxiety via need frustration.
Barkoukis et al. (2010) (38)	Greece	317; 53.6%; 13.9 ± 0.76	LAPOPECQ	State anxiety; Self-efficacy	PESAS	TARGET intervention improved motor skill self-efficacy but did not reduce state anxiety.
Li et al. (2024) (39)	USA	255; 49.8%; 18.87 ± 0.92	PMCSQ-2	Perceived stress; Psychological distress; Social dysfunction	PSS; GHQ-12	Sport Education Model enhanced task-involving climate, intrinsic motivation, and reduced social dysfunction vs. traditional PE; both groups reduced global stress.
Coterón López et al. (2013) (40)	Spain	1,587; 48.65%; 15.26 ± 1.61	Spanish-adapted PMCSQ-2	PE anxiety	AMPET	Ego climate predicted higher anxiety and perceived competence; task climate predicted higher commitment. Compulsory students reported higher anxiety than post-compulsory peers.
Vlachos et al. (2025) (41)	Greece	979; 47.9%; 13.75 ± 1.96	MUMOC-PES	PE anxiety; Class satisfaction	SAS	Perceived empowering climate predicted higher satisfaction and lower anxiety; disempowering/controlling climate predicted higher anxiety.

Sport climate measures: MCPEQ, Motivation Climate in Physical Education Questionnaire; PE-EMCS, PE Empowering Motivational Climate Scale; EMCS, Empowering motivational climate scale; PMCEQ-A, Perceived Motivational Climate in Exercise Questionnaire–Abbreviated; CCS, Caring Climate Scale; PMCSQ, Perceived Motivational Climate in Sport Questionnaire; PMCSQ-2, Perceived Motivational Climate in Sport Questionnaire–2; PNSS-S, Psychological Need States in Sport Scale; MCSYS, Motivational Climate Scale for Youth Sports; CART-Q, Coach–Athlete Relationship Questionnaire; YSEQ, Youth Sport Environment Questionnaire; EDMCQ-PE, Empowering and Disempowering Motivational Climate Questionnaire in Physical Education; TIMCPEQ, Teacher-Initiated Motivational Climate in Physical Education Questionnaire; TASCQ, Teacher as Social Context Questionnaire; Peer MCYSQ, Peer Motivational Climate in Youth Sport Questionnaire; MCPES, Motivational Climate in Physical Education Scale; PACES, Physical Activity Enjoyment Scale; STAI, State-Trait Anxiety Inventory; LAPOPECQ, Learning and Performance-Oriented Physical Education Climate Questionnaire; MUMOC-PES, Multidimensional Motivational Climate Questionnaire in Physical Education at the Situational Level of Generality.

Mental health measures: PSS, Perceived Stress Scale; ISS, Internalised Shame Scale; PASA, Primary Appraisal Secondary Appraisal Scale; BDI-PC, Beck Depression Inventory for Primary Care; PROMIS, Patient-Reported Outcomes Measurement Information System; Campbell Happiness Index, subjective well-being; Sport Enjoyment Scale, physical education enjoyment; DSECS-S, Delaware Social-Emotional Competency Scale; Rosenberg Self-Esteem Scale, global self-esteem; Life Skills Scale for PE, life skills in physical education; PESAS, Physical Education State Anxiety Scale; PBS, psychobiosocial states; PSDQ, Physical Self-Description Questionnaire; SCQ-2, Sport Commitment Questionnaire–2; PANAS, Positive and Negative Affect Schedule; GHQ12 = 12-item General Health Questionnaire; AMPET, Achievement Motivation in Physical Education Test; SAS, Sport Anxiety Scale; adapted Classroom Satisfaction Scale, adapted Physical Education Classroom Satisfaction Scale.

### 3.4 Data synthesis

#### 3.4.1 Motivational climate and positive mental well-being

Across the included studies examining positive mental well-being indicators, supportive and empowering sport climates were generally associated with more favourable psychological outcomes amongst adolescents. Specifically, sport and physical education environments characterised by competence support, positive interpersonal interactions, and empowering motivational features were related to higher levels of subjective well-being, physical self-concept, and positive affect.

Evidence regarding global well-being indicators from four studies examining this domain consistently found positive associations between supportive sport environments and adolescentss psychological functioning. Campus sport atmosphere was positively associated with subjective well-being (e = 0.289, p <.01), with peer relationships and positive emotions identified as mediating pathways (23). Similarly, empowering climates were associated with more favourable self-related perceptions, with competence satisfaction representing a potential mediating pathway (a = 0.52, p <.001) (9).

Longitudinal evidence further indicated a temporal association between supportive educational climates and adolescentsl self-related perceptions. Teacher need support within physical education was positively associated with stable physical self-concept over time (b = 0.17, p <.01), whilst autonomous motivation was also positively associated with physical self-concept (b = 0.16, p <.05) (31). These findings suggest an association between supportive educational climates and more stable self-perceptions during adolescence.

Positive affective indicators were also examined in relation to empowering climates. Empowering motivational climate was associated with competence need satisfaction (a = 0.43, p <.05), which was further associated with higher positive affect (f = 0.66) and lower negative affect (f = −0.19) (22). Overall, available evidence indicates that supportive and empowering sport climates are associated with more favourable positive psychological outcomes amongst adolescents.

#### 3.4.2 Motivational climate and negative psychological indicators

Across the included studies examining negative psychological indicators, maladaptive motivational climates characterised by ego orientation, performance comparison, and psychological need frustration were generally associated with poorer psychological outcomes amongst adolescents. Conversely, task-involving, caring, and autonomy-supportive climates were associated with lower levels of psychological distress, including stress, anxiety, shame, and depressive symptoms, across sport and physical education settings.

Stress and shame were examined in three studies, all of which reported associations in the expected direction. Task-oriented climates were negatively associated with perceived stress, whereas ego-oriented climates showed positive associations with stress amongst adolescents of both genders (task climate: boys β = −0.300, girls β = −0.121; ego climate: boys β = 0.105, girls β = 0.145) (6). Similarly, ego climates were associated with higher life stress and shame, whereas caring climates were associated with lower shame amongst adolescents (8). Further evidence indicated that caring climates were negatively associated with depression, life stress, shame, and psychosocial stress (β = −0.26 to −0.44) (21).

Anxiety-related outcomes showed a comparable pattern. Ego-oriented climates were associated with higher anxiety symptoms, whereas task-oriented and empowering climates demonstrated more favourable associations. Specifically, ego climate was related to greater anxiety across somatic, cognitive, and worry dimensions (20), whilst task climate was associated with lower anxiety through competence-related pathways (β = −0.074 to −0.141) (32). Moreover, empowering climates were negatively associated with state anxiety (r = −0.29, p <.01), whereas disempowering climates were associated with increased anxiety through psychological need frustration (β = 0.23, p <.01) (24). Extended evidence from a longitudinal intervention also indicated that both Sport Education and traditional PE groups reduced perceived stress and psychological distress over time (39). Additionally, evidence from self-report and observational studies indicated that controlling teacher behaviours and ego-involving climates were associated with higher student anxiety (40, 41).

Evidence from a large youth sport sample further extended these findings to depressive and anxiety symptoms. Ego climate was positively associated with depression (β = 0.205) and anxiety symptoms (β = 0.184), whereas autonomy support, coach closeness, and social cohesion were negatively associated with these outcomes (26). Collectively, climates characterised by comparison, external evaluation, and psychological need frustration were associated with greater psychological distress, including stress, anxiety, shame, and depressive symptoms.

#### 3.4.3 Motivational climate and affective and motivational outcomes

Across studies examining affective and motivational outcomes, task-involving and empowering climates were generally associated with more favourable emotional experiences, whereas ego-involving and disempowering climates were linked to reduced enjoyment, increased anxiety, and dysfunctional psychobiosocial states.

Enjoyment was examined in four studies, all of which reported positive associations with task-involving and learning-oriented climates. Learning- and task-oriented climates were associated with higher enjoyment and lower anxiety across longitudinal and cross-sectional designs (19, 20, 32). Specifically, learning-oriented climate predicted physical education enjoyment directly (β = 0.41 and 0.40, p <.001) and showed a statistical indirect association through intrinsic motivation (β = 0.21–0.27) (19). Task climates were associated with greater enjoyment (β = 0.48–0.53) and lower anxiety (β = −0.074 to −0.141), whereas ego climates were associated with reduced enjoyment (β = −0.11) and higher anxiety across somatic, cognitive, and worry dimensions (β = 0.14–0.37; β = 0.014–0.032) (20, 32).

Evidence from empowering climate studies showed similar patterns. Similarly, empowering climates were associated with greater pleasure through psychological need satisfaction (β = 0.51), whereas disempowering climates were associated with higher state anxiety through need frustration (β = 0.23) (24). Experimental evidence further showed that ego-involving climates reduced pleasant and functional psychobiosocial states (d = −0.33) and increased unpleasant and dysfunctional states (d = 0.57) (25).

Overall, task-involving and empowering climates were associated with more adaptive affective experiences, whereas ego-involving and disempowering climates showed less favourable emotional patterns.

#### 3.4.4 Motivational climate and psychosocial and developmental competencies

Across studies examining psychosocial and developmental competencies, empowering and supportive motivational climates were generally associated with higher levels of social-emotional competence, life skills, coping appraisals, self-esteem and physical self-concept amongst adolescents.

Empowering climates were positively associated with adolescents’ developmental competencies. Specifically, empowering climates predicted higher social-emotional competence (β = 0.19, p <.01), with teacher–student relationships and physical activity contributing to this association (29). Similarly, empowering climates were positively related to self-esteem through basic psychological need satisfaction (β = 0.16, p <.001), whereas disempowering climates were negatively associated with self-esteem (β = −0.22, p <.001) (9). Additionally, task- and caring-oriented climates were associated with stronger coping appraisals, whereas ego climates showed less adaptive coping-related responses amongst adolescents (8, 21).

Evidence regarding broader developmental indicators showed comparable patterns. Task-involving climate interventions were also associated with enhanced self-efficacy in physical education settings (38). Empowering climates were positively associated with life skills in physical education, including teamwork, goal setting, communication, and emotional skills (β = 0.329, p <.001; β = 0.656) (27). Longitudinal evidence further indicated that teacher need support (b = 0.17, p <.01) and autonomous motivation (b = 0.16, p <.05) were positively associated with adolescents’ physical self-concept over time (31).

Collectively, empowering and supportive climates were associated with adolescentsy broader developmental competencies, including self-esteem, coping resources, life skills, and social-emotional competence.

## 4 Discussion

### 4.1 Summary of main findings

Overall, this review identified a broadly consistent pattern whereby task-involving, learning-oriented, caring, and empowering motivational climates were associated with more favourable psychological outcomes, whereas ego-involving and disempowering climates were associated with greater psychological distress across multiple mental health domains. Importantly, the influence of school sport climate extended beyond traditional motivational outcomes to encompass adolescents’ psychological well-being, emotional experiences, psychological distress, and broader psychosocial competencies.

The findings can be interpreted through the complementary perspectives of AGT and SDT. From an AGT perspective, task-involving climates emphasise effort, self-improvement, and mastery rather than normative comparison, thereby promoting perceptions of competence and reducing concerns related to failure and evaluation (5). Conversely, ego-involving climates emphasise outperforming others and demonstrating ability, which may increase anxiety, shame, and stress, particularly amongst adolescents with lower perceived competence (25, 28, 40). Consistent with SDT, supportive sport climates facilitate satisfaction of autonomy, competence, and relatedness needs, which subsequently promote intrinsic motivation and psychological well-being (7). Across the reviewed studies, basic psychological need satisfaction and intrinsic motivation emerged as important explanatory pathways linking motivational climate with adolescent mental health outcomes (9, 24).

Beyond motivational mechanisms, the findings suggest that cognitive and self-regulatory processes may also contribute to understanding how sport-related experiences are associated with adolescent psychological functioning. Recent evidence amongst sports high-school adolescents indicated that ruminative thinking was associated with subjective well-being, with cognitive-behavioural physical activity demonstrating a statistical indirect role in this association (34). Although such indirect effects should not be interpreted as causal mechanisms, these findings complement the present review by highlighting the potential relevance of cognitive appraisal and self-regulatory processes in explaining pathways linking sport experiences with psychological outcomes.

The present review further indicates that school sport climate should be conceptualised as a broader social-ecological context rather than merely a motivational environment. Interpersonal factors, including teacher–student relationships, coach closeness, peer acceptance, team cohesion, and supportive interactions, were consistently associated with better mental health outcomes (21, 23, 26, 29). These relational factors correspond closely with adolescents’ developmental needs for social connection, belonging, and identity formation. During adolescence, when individuals are particularly sensitive to social acceptance and interpersonal experiences, supportive sport environments may provide opportunities for connection, recognition, and psychological safety (1, 4). Conversely, experiences of criticism, exclusion, excessive competition, and interpersonal conflict may undermine adolescents’ sense of belonging and increase psychological vulnerability (8, 28). Thus, the association between school sport climate and adolescent mental health appears to operate through both motivational and relational pathways.

Although broadly consistent patterns were observed across cultural contexts, some contextual variations were identified. Some studies from collectivistic contexts (e.g., China) have highlighted the role of teacherhtedtic3 relationships, whereas studies from Western contexts more frequently highlighted peer cohesion and coach–athlete relationships (8, 26, 29). These differences may reflect variations in cultural values related to authority, interdependence, and social relationships. However, given the limited number of studies available within individual cultural contexts, these interpretations remain preliminary and warrant further investigation through cross-cultural research designs.

This review extends previous literature by reconceptualising school sport climate as an important psychosocial context for adolescent mental health, rather than merely a determinant of motivation, engagement, or participation outcomes (4, 12, 13, 30). The findings suggest that sport environments characterised by supportive interactions, autonomy support, and mastery-oriented values may provide adolescents with opportunities to experience psychological safety, competence development, and social connection. Given the central role of physical education and school sport in adolescents, everyday lives, creating supportive sport climates may represent a feasible and scalable strategy for integrating mental health promotion into educational settings (3, 6, 10).

### 4.2 Limitations

There are some caveats to interpreting the findings. For one, the majority of studies included used a cross-sectional design, meaning that causal and temporal relationships could not be drawn from the identified associations, since adolescents’ perceptions of sport climate may also affect their mental health. Second, there was significant heterogeneity in the way motivational climate and mental health outcomes were measured and some studies lacked formal mediation analyses or provided only associations, which inhibited the interpretation of possible pathways. Furthermore, there is conceptual overlap between the empowering and task-involving climates and also between the disempowering and ego-involving climates, which may make the empirical differentiation of these climates difficult (30). Third, methodological quality was judged using JBI tools but the overall certainty of evidence was not formally judged and the strength of the synthesised findings should be interpreted with caution. Fourth, only specific databases were searched and only English-language publications were included, and other studies that were not in English and grey literature may not have been found, thus introducing selection bias. Finally, some of the demographic data was reported inconsistently, making it difficult to perform some of the meta analyses.

### 4.3 Strengths

There are strength to this review, notwithstanding these limitations. First, it was a review conducted in keeping with PRISMA guidelines and used a systematic approach in identifying, screening, data extraction, and methodological quality appraisal, ensuring transparency and reproducibility. Second, this review included a wide variety of adolescent mental health indicators such as positive well-being, emotional experiences, psychological distress, and psychosocial competencies, which allows for a more holistic understanding of the implications of school sport climate, unlike other reviews that have mostly focused on motivational or behavioural outcomes. Thirdly, this review, informed by Achievement Goal Theory, Self-Determination Theory and social-ecological frameworks, revealed a number of psychological and relational mechanisms by which motivational climates might be related to mental health in adolescence. Lastly, the use of studies across different cultural contexts enriches the context of the findings, at the same time pointing to the need for more cross-cultural studies.

### 4.4 Practical and policy implications

The findings of this review offer several evidence-informed implications for PE teachers, school administrators, and education policymakers. At the classroom level, PE teachers may consider fostering task-involving, caring, and empowering motivational climates to support adolescents’ psychological functioning. Teaching and evaluation practices could emphasise individual effort, self-improvement, and learning progress rather than excessive competition and normative comparison. Teachers may also benefit from incorporating autonomy-supportive strategies, constructive feedback, and approaches that enhance students’ perceived competence and positive interpersonal experiences. For school administrators, providing professional development opportunities for PE teachers may be a promising approach to strengthen supportive teaching practices, communication skills, and awareness of students’ psychological needs. For education policymakers, the findings suggest that school sport policies could consider promoting health-oriented and inclusive sport cultures whilst balancing competitive evaluation systems with students’ psychological well-being. Optimising motivational climate in school physical education and sport may represent a feasible and scalable target for school-based mental-health promotion; however, its effectiveness requires confirmation through well-designed longitudinal and intervention studies, particularly those examining causal effects and implementation outcomes.

### 4.5 Recommendations for future research

Several directions are suggested for the future research for the advancement of the research in this field. To unravel the causal mechanisms underlying the link between sport climate and mental health, longitudinal designs and intervention trials are needed. To minimise the diversity of measurement instruments and to allow for quantitative meta-analysis, standardised measurement instruments should be developed. Additional multi group analyses will assist in identifying at risk groups and in developing targeted interventions. Mixed-methods can add to the depth of understanding of the “real-life” experiences of adolescents in sport settings. The scope of future research could also be expanded to out of school sports and multi-level environmental influences, such as family and peer groups.

## 5 Conclusion

Adolescent mental health is a global public health priority, and school PE and sport are irreplaceable venues for mental health promotion. This systematic review demonstrates that supportive, mastery-oriented motivational climates benefit adolescents’ psychological well-being, whereas ego-involving and disempowering climates increase psychological risks. Multiple psychological, social, cognitive, and behavioural pathways may contribute to these associations, although the available evidence does not allow causal conclusions. In the interest of the protection and promotion of adolescent mental health, schools and teachers should collaborate and jointly aim to optimise the sport and PE environment for a supportive atmosphere. Hopefully the synthesised evidence will help inform practical interventions and inspire further in-depth research in this important field.

## Data Availability

The original contributions presented in the study are included in the article/**Supplementary Material**. Further inquiries can be directed to the corresponding author.
