# Supplementary material for: Motivational climate in school sport and adolescent mental health: a systematic review
Source: Front Psychiatry. 2026 Sep 3;17:1907748. doi: 10.3389/fpsyt.2026.1907748 (PMC13582401; doi:10.3389/fpsyt.2026.1907748)
Supplement: Supplementary file 1 [file Table1.docx]

**Supplementary Material S1**

Web of Science = 54

TS = ("motivational climate" OR "task-involving climate" OR "task climate" OR "mastery climate" OR "mastery-oriented climate" OR "ego-involving climate" OR "ego climate" OR "performance climate" OR "performance-oriented climate" OR "caring climate" OR "empowering climate" OR "disempowering climate"

OR "achievement goal climate") AND ("physical education" OR "physical education class*" OR "school sport*" OR "school-based sport*" OR "school athletics" OR "athletic team*" OR "intramuralsport*") AND ("mental health" OR wellbeing OR "well-being" OR "subjective well-being" OR "psychological well-being" OR "lifesatisfaction" OR "self-esteem" OR anxiety OR depression OR stress OR shame OR "psychological distress" OR "positive affect" OR "negative affect" OR "social-emotional competence")

PubMed = 45

MeSH Terms= ("motivational climate" OR "task-involving climate" OR "task climate" OR "mastery climate" OR "mastery-oriented climate" OR "ego-involving climate" OR "ego climate" OR "performance climate" OR "performance-oriented climate" OR "caring climate" OR "empowering climate" OR "disempowering climate"

OR "achievement goal climate") AND ("physical education" OR "physical education class*" OR "school sport*" OR "school-based sport*" OR "school athletics" OR "athletic team*" OR "intramuralsport*") AND ("mental health" OR wellbeing OR "well-being" OR "subjective well-being" OR "psychological well-being" OR "lifesatisfaction" OR "self-esteem" OR anxiety OR depression OR stress OR shame OR "psychological distress" OR "positive affect" OR "negative affect" OR "social-emotional competence")

Sage Journals =246

Anywhere= ("motivational climate" OR "task-involving climate" OR "task climate" OR "mastery climate" OR "mastery-oriented climate" OR "ego-involving climate" OR "ego climate" OR "performance climate" OR "performance-oriented climate" OR "caring climate" OR "empowering climate" OR "disempowering climate"

OR "achievement goal climate")

AND

("physical education" OR "physical education class*" OR "school sport*" OR "school-based sport*" OR "school athletics" OR "athletic team*" OR "intramuralsport*")

AND

("mental health" OR wellbeing OR "well-being" OR "subjective well-being" OR "psychological well-being" OR "lifesatisfaction" OR "self-esteem" OR anxiety OR depression OR stress OR shame OR "psychological distress" OR "positive affect" OR "negative affect" OR "social-emotional competence")

Springer Nature Link =183

Keywords= ("motivational climate" OR "task-involving climate" OR "task climate" OR "mastery climate" OR "mastery-oriented climate" OR "ego-involving climate" OR "ego climate" OR "performance climate" OR "performance-oriented climate" OR "caring climate" OR "empowering climate" OR "disempowering climate"

OR "achievement goal climate") AND ("physical education" OR "physical education class*" OR "school sport*" OR "school-based sport*" OR "school athletics" OR "athletic team*" OR "intramuralsport*") AND ("mental health" OR wellbeing OR "well-being" OR "subjective well-being" OR "psychological well-being" OR "lifesatisfaction" OR "self-esteem" OR anxiety OR depression OR stress OR shame OR "psychological distress" OR "positive affect" OR "negative affect" OR "social-emotional competence")

EBSCOhost (SPORTDiscus, PsycINFO, ERIC) = 54

AB= ("motivational climate" OR "task-involving climate" OR "task climate" OR "mastery climate" OR "mastery-oriented climate" OR "ego-involving climate" OR "ego climate" OR "performance climate" OR "performance-oriented climate" OR "caring climate" OR "empowering climate" OR "disempowering climate" OR "achievement goal climate") AND ("physical education" OR "physical education class*" OR "school sport*" OR "school-based sport*" OR "school athletics" OR "athletic team*" OR "intramuralsport*") AND ("mental health" OR wellbeing OR "well-being" OR "subjective well-being" OR "psychological well-being" OR "lifesatisfaction" OR "self-esteem" OR anxiety OR depression OR stress OR shame OR "psychological distress" OR "positive affect" OR "negative affect" OR "social-emotional competence")
